# The Long Non-coding RNA MEG3/miR-let-7c-5p Axis Regulates Ethanol-Induced Hepatic Steatosis and Apoptosis by Targeting NLRC5

**DOI:** 10.3389/fphar.2018.00302

**Published:** 2018-04-10

**Authors:** Qin Wang, Mingfang Li, Zhiming Shen, Fangtian Bu, Haixia Yu, Xueyin Pan, Yang Yang, Xiaoming Meng, Cheng Huang, Jun Li

**Affiliations:** ^1^Anhui Province Key Laboratory of Major Autoimmune Diseases, Anhui Institute of Innovative Drugs, School of Pharmacy, Anhui Medical University, Hefei, China; ^2^The Key Laboratory of Anti-inflammatory and Immune Medicines, Ministry of Education, Hefei, China; ^3^Department of Cardiac Surgery, First Affiliated Hospital of Anhui Medical University, Hefei, China

**Keywords:** lncRNA, MEG3, hepatic steatosis, apoptosis, miR-let-7c-5p, NLRC5

## Abstract

Ethanol (EtOH)-induced hepatic injury, characterized by hepatic steatosis with apoptosis, causes heavy health burden personally and socially. Long non-coding RNAs (lncRNAs) have been implicated in liver diseases. However, the role of lncRNA maternally expressed gene 3 (MEG3) in EtOH-induced hepatic injury remains unknown. The aim of present study was to assess the function of MEG3 and its functional interaction with miR-let-7c-5p in EtOH-induced hepatic injury. Here, we observed that MEG3 and NLRC5 expression was increased and miR-let-7c-5p expression decreased in EtOH-fed mice and EtOH-induced AML-12 cells. Knockdown of MEG3 contributed to attenuation of EtOH-induced steatosis and apoptosis in AML-12 cells. Also, expression level of MEG3 negatively correlated with miR-let-7c-5p expression and positively correlated with NLRC5 expression. In contrary to MEG3, miR-let-7c-5p overexpression attenuated EtOH-induced steatosis and apoptosis, as well as suppressed EtOH-induced increase in NLRC5 expression. By luciferase reporter assay, we concluded that miR-let-7c-5p directly binds to NLRC5 3′-UTR, thereby negatively regulates NLRC5 expression. Our data suggested that lncRNA MEG3 functions as a competing endogenous RNA for miR-let-7c-5p to regulate NLRC5 expression in EtOH-induced hepatic injury.

## Introduction

Alcoholic liver disease (ALD) is associated with too much alcohol consumption and often presents after 20–30 years chronic abuse alcohol which is one of the predominant causes of liver-related morbidity and mortality worldwide (Dubuquoy, 2016). It includes a broad clinical-histological from alcoholic steatosis, alcoholic hepatitis, cirrhosis to hepatocellular carcinoma. About 20% of alcoholics develop into ALD (Gao and Bataller, 2011). Alcoholic steatosis is characterized by increased accumulation of lipids, triglyceride, and cholesterol. Meanwhile, alcohol consumption also results in hepatocyte apoptosis. (Zeng and Xie, 2009; Gao and Bataller, 2011). Alcoholic steatosis plays a significant role in preventing the evolution of ALD toward severe stages (Bieghs et al., 2013). However, the molecular mechanisms underlying EtOH-induced hepatic steatosis and apoptosis activity remains largely unknown.

Recently, studies have revealed that more than 90% of the human genome is transcribed, but most of the transcripts are referred to as non-coding RNAs (ncRNAs) (Esteller, 2011). Among them, long non-coding RNAs (lncRNAs) are one kind of endogenous RNA comprises a sequence more than 200 nucleotides (Chen, 2016). Many lncRNAs are known to play important roles in many biological processes, such as cell development, cell differentiation, and cell apoptosis (Qureshi et al., 2010; Gibb et al., 2011; Rinn and Chang, 2012; Fatica and Bozzoni, 2014). So far, lncRNAs have been demonstrated to regulate gene expression at both the transcriptional and posttranscriptional levels (Chen, 2016). It has been reported that the lncRNAs have been shown to play significant effects on several liver diseases, such as hepatic fibrosis and hepatocellular carcinoma (Bian et al., 2017; Hu et al., 2017). Maternally expressed gene 3 (MEG3) is an lncRNA with a length of ∼1.6 kb nucleotides and is reciprocally imprinted with the paternally expressed gene DLK1 constituting an imprinting domain on human chromosome 14q32, with its homolog of mouse MEG3 on the distal region of mouse chromosome 12 (Miyoshi et al., 2000). MEG3 have a significant role in mice normal growth and survival (Takahashi et al., 2009; Stadtfeld et al., 2010). In humans, MEG3 is expressed in many normal tissues, while the loss of expression has been found in various types of tumor tissues and cell lines (Li L. et al., 2017; Li Z.H. et al., 2017; Terashima et al., 2017). However, the mechanism underlying the role of MEG3 in EtOH-induced hepatic steatosis and apoptosis is inadequate.

Recent studies proposed a viewpoint that lncRNA can suppress messenger RNA (mRNA) translation by microRNA (miRNA), resulting in increased expression of corresponding protein (Cai et al., 2017; Wang et al., 2017; Zhao et al., 2017). MEG3 has been described to be a ceRNA that regulates miR-223 (Zhang Y. et al., 2017), miR-183 (Zhang and Feng, 2017), miR-421 (Zhang W. et al., 2017), and miR-127 (Wang and Kong, 2018). miRNAs are a class of small ncRNAs that are typically 20–22 nucleotides in length (Bartel, 2009). The let-7 miRNA family is one of the first two miRNAs discovered in *Caenorhabditis elegans*, and the first known human miRNA, consisting of let-7a, b, c, d, e, f, i and miR-98 miRNAs (Roush and Slack, 2008). The let-7 miRNA family was found to play an important role in liver diseases (Matsuura et al., 2016; McDaniel et al., 2017).

Nucleotide-binding domain and leucine-rich repeat containing receptors (NLRs) represent a large family of intracellular pattern recognition receptors (PRRs) that are characterized by a conserved nucleotide-binding and oligomerization domain (NOD) and a leucine-rich repeat (LRR) region (Abrahams, 2011). NLRC5 is a newly identified member of the NLR protein family and has recently been identified as a critical regulator of immune responses (Kobayashi and van den Elsen, 2012; Downs et al., 2016). Previous studies from our laboratory have proved that NLRC5 plays a negative role in ethanol-induced hepatic steatosis. We also demonstrated that NLRC5 promoted proliferation and activation of hepatic stellate cells during hepatic fibrosis (Liu et al., 2016b).

In this study, we found that lncRNA MEG3 was increased in EtOH-fed mice and EtOH-induced AML-12 cells. We demonstrated that MEG3 could directly bind to miR-let-7c-5p and effectively act as a sponge for miR-let-7c-5p to modulate the suppression of NLRC5. The MEG3/miR-let-7c-5p/NLRC5 axis functions as an important player in EtOH-induced hepatic steatosis and apoptosis.

## Materials and Methods

### Mouse Model

All animal experiments were approved by the Anhui Medical University Animal Care and Use Committee and were conducted in accordance with National Institutes of Health guidelines. 8-week-old male C57BL/6J mice were purchased from the Experimental Animal Center of Anhui Medical University. The mice were randomly divided into control diet (CD)-fed group and EtOH-fed group.

A mouse model of chronic plus single binge ethanol gavage was used as described (Bertola et al., 2013). For chronic alcohol administration, the first 5 days, all mice were fed a liquid control diet, then EtOH-fed mice were fed a liquid diet containing 5% w/v ethanol for 10 days, whereas the CD-fed mice were fed with isocaloric maltose-dextrin. At day 16, EtOH-fed mice were given a single dose of ethanol gavage (5 g/kg body weight, 20% ethanol), while CD-fed mice were given an isocaloric dose of dextrin maltose gavage. 9 h post-gavage, the mice were euthanized and blood and tissue samples were collected for the further analysis.

### Isolation of Primary Hepatocytes

Hepatocytes were isolated from male C57 BL/6 mice as previously described (Morris et al., 2012). Primary hepatocytes isolated from individual animals were frozen at -80°C for each experiment.

### Biochemical Analysis

Enzyme activities of aspartate aminotransferase (AST), alanine aminotransferases (ALTs), triglyceride (TG), and total cholesterol (TCH) were detected using commercial assay kits according to the protocols from manufacturer (Nanjing Jiancheng Bioengineering Institute, Nanjing, China). The absorbance values at 510 nm were obtained with a micro-plate reader model 680 (Bio-Rad Laboratories, Hercules, CA, United States).

### Histopathological Analysis

Liver tissues were fixed in 4% formalin for 24 h and embedded in paraffin, and wax were sliced and toasted for 1 h at 60°C. Slides were dewaxed in xylene and dehydrated in alcohol, then antigen retrieval was achieved by microwaving in citric saline for 15 min. The sections were treated with 0.3% hydrogen peroxide for 15 min to block endogenous peroxidase activity. Then the sections were blocked by 5% BSA (Bovine Serum Albumin) (37°C, 20 min) and conjugated with primary antibody against NLRC5 (4°C, 12 h) and biotinylated secondary antibody (37°C, 60 min). NLRC5 was visualized by 3,3′-diaminobenzidine tetrahydrochloride (DAB) staining. Then, the sections were hematoxylin-redyed and dehydrated in gradient ethanol (30, 50, 70, 80, 90, 95, and 100% for 3 min, respectively) and xylene for 20 min. Finally, the sections were mounted by gums and subjected to microscopic examination. Fresh liver tissues were immersed in freezing medium and stored at -80°C. Then, the liver tissues were sliced, washed by PBS for three times (5 min each time) and stained with Oil Red O according to the protocols from manufacturer (Nanjing Jiancheng Bioengineering Institute, Nanjing, China).

### Flow Cytometry Analyses of Apoptosis

Apoptosis was detected by fluorescein isothiocyanate (FITC)-labeled Annexin-V/propidium iodide (PI) double staining and flow cytometry analysis. AML-12 cells were collected from suspension by centrifugation. An Annexin-FITC apoptosis assay kit was used in strict accordance to the protocol from the manufacturer (BestBio, China). When the preparation was completed, cells suspension was detected by flow cytometer (BD Biosciences) and data fitting was made by FlowJo software (Tree Star, United States). All samples were repeated three times.

### Cell Culture and Cell Treatment

Mouse hepatocyte AML-12 cells were purchased from Cell Bank of Chinese Academy of Sciences (Shanghai, China). Cells were maintained in Dulbecco’s Modified Eagle’s Medium (DMEM), supplemented with 10% heat-inactivated fetal bovine serum (FBS) (Every Green, China) and incubated at 37°C at an atmosphere of 5% CO_2_. AML-12 cells were transiently transfected with MEG3 small interfering RNA (siRNA) or miR-let-7c-5p mimics/inhibitor and NC (GenePharma, Shanghai, China) with Lipofectamine 2000 reagent (Invitrogen, Carlsbad, CA, United States) according to the manufacturer’s protocol.

### Quantitative Real-Time PCR

Total RNA was extracted from mouse primary hepatocytes and AML-12 cells using Trizol reagents (Invitrogen, United States). One microgram of total mRNA was used to reverse transcribe cDNA with Takara RT-PCR synthesis kit, according to the manufacturer’s instructions (TAKARA, Dalian, China). The cDNA was performed using SYBR Premix Ex Taq II (TAKARA, Dalian, China) on the PikoReal 96 qPCR system (Thermo Scientific, United States). The mRNA level of GAPDH was relative to internal control. The primer sequences (Sangon Biotech, China) which we used were as following: GAPDH (forward: 5′-AACGGATTTGGCCGTATTGG-3′; reverse: 3′-CATTCTCGGCCTTGACTGTG-5′), MEG3(forward: 5′-ATACACACATTGGGCCTCCA-3′; reverse: 3′-ACAGCCCATGGTATCACACA-5′), PPAR-α (forward: 5′-TTCCTGCCACTTGCTCACTA-3′; reverse: 3′-TTCACCCTGATTCCTGATGTC-5′), SREBP-1c (forward: 5′-TGTCTGGGAAGGGAGCATAA-3′; reverse: 3′-GCTGTTCTGTGGTTTGTTTACCT-5′), NLRC5 (forward: 5′-CTCAGCAGGAATGGCTTGTC-3′; reverse: 3′-ATGCAGTCACTCTCAAGGCT-5′’), fatty acid synthase (Fasn) (forward: 5′-AAGTTGCCCGAGTCAGAGAA-3′; reverse: 3′-TTCCAGACCGCTTGGGTAAT-5′), acetyl CoAcarboxylase (Acaca) (forward: 5′-ATGAAGGCTGTGGTGATGGA-3′; reverse: 3′- CATTTGTCGTAGTGGCCGTT-5′). For miR-let-7c-5p, the procedure was performed according to the manuscript of the one-step miRNA qRT-PCR Detection Kit (Biomics, Nantong, Jiangsu, China), and U6 snRNA was used as the endogenous control. All experiments were repeated at least three times.

### Western Blot Analysis

Total proteins were extracted with Radio-Immunoprecipitation Assay (RIPA) buffer including phenylmethanesulfonyl fluoride (PMSF) (Beyotime, China). Whole extracts were prepared, and protein concentration was detected using a BCA protein assay kit (Beyotime, China). Total protein from samples were separated by SDS-PAGE and blotted onto the PVDF membrane (Millipore, Billerica, MA, United States) and then blocked with 5% skimmed milk for 2 h. The transferred membranes were incubated with primary antibody overnight at 4°C. After three washes in TBS/Tween-20, blots were incubated for 1 h in goat anti-mouse or goat anti-rabbit horseradish peroxidase (HRP, Santa Cruz, CA, United States) conjugate antibody at 1:10000 dilution in TBS/Tween-20 containing 5% skimmed milk. The membranes were also washing three times with TBS/Tween-20, protein blots were detected by ECL-chemiluminescent kit (Thermo Scientific, United States). The categories and concentrations of primary antibodies: anti-PPAR-α (Rabbit, 1:800, ab8934, Abcam, United Kingdom), SREBP-1c (Rabbit, 1:800, ab28481, Abcam, United Kingdom), anti-Bax (Rabbit, 1:800, ab32503, Abcam, United Kingdom), anti-Bcl2 (Rabbit, 1:800, ab196495, Abcam, United Kingdom), anti-caspase 9 (Rabbit, 1:800, ab184786, Abcam, United Kingdom), and anti-NLRC5 (Rabbit, 1:800, ab117624, Abcam, United Kingdom); anti-caspase 3 (Rabbit, 1:800, #8242, Cell Signaling Tech, Danvers, MA, United States), anti-β-actin (Mouse, 1:300, sc47778, Santa Cruz, CA, United States). All experiments were repeated three times.

### Luciferase Reporter Assays

293T cells were co-transfected with the wild-type (wt-MEG3, wt-NLRC5 -3′UTR)/mutated (mut-MEG3, mut-NLRC5-3′UTR) and miR-let-7c-5p mimics. Luciferase activities were measured at 48 h after co-transfection with the Dual-Luciferase Reporter Assay System (Promega).

### LncRNA Fluorescence *in Situ* Hybridization (FISH)

The expression and localization of MEG3 were determined by lncRNA FISH in liver tissues according to the instructions of the Fluorescent *In Situ* Hybridization Kit (RiboBio, Guangzhou, China). After formaldehyde fixation, tissues were pre-hybridized for 30 min at 37°C and then hybridized for 12 h at 37°C with a 1:50 dilution of lncRNA FISH Probe Mix provided by the kit. After washing, the tissues were stained with DAPI for 5 min and images were taken using inversion fluorescence microscopy.

### Statistical Analysis

The experimental data were evaluated by calculating the mean ± SD. Differences between multiple groups were evaluated using one-way analysis of variance. Differences between two groups were compared using a Student’s *t*-test. The significance level was set at *P* < 0.05.

## Results

### Ethanol Consumption Produces Liver Injury, Steatosis, and Apoptosis

**FIGURE 1:**
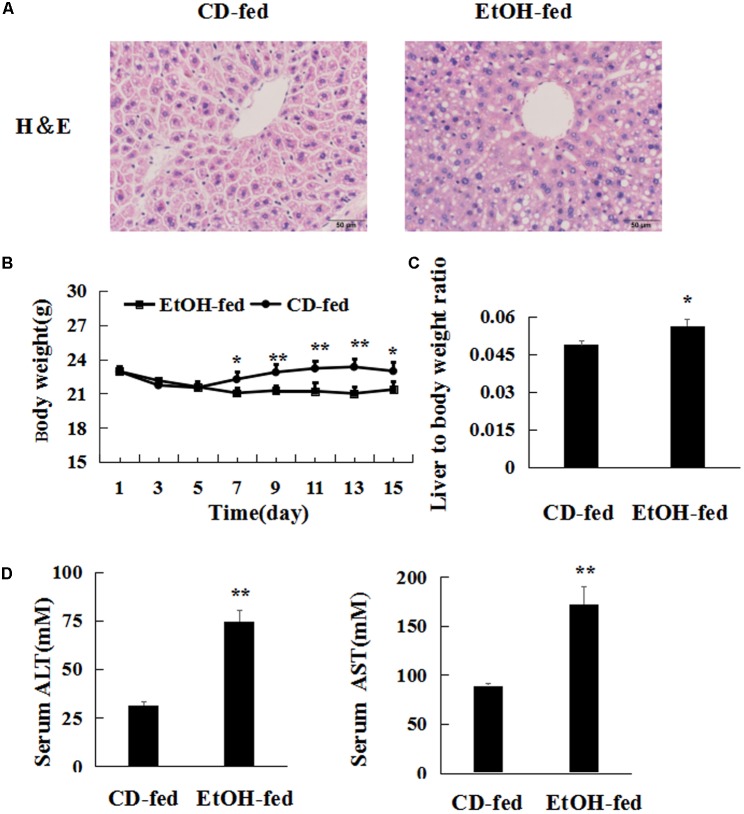
Chronic ethanol consumption produces injury. **(A)** HE stained liver of alcohol-treated mouse and control mouse 400×. **(B)** Body weights of EtOH-fed mice and CD-fed mice. **(C)** Liver to body weight ratio after ethanol feeding. **(D)** Serum ALT and AST levels. The values represent means ± SD (*n* = 6 in CD-fed group, *n* = 6 in EtOH-fed group). ^∗^*P* < 0.05, ^∗∗^*P* < 0.01 vs. CD-fed group.

Mice with chronic alcohol feeding were characterized by injury in the liver. To investigate the degree of liver injury produced by ethanol consumption, we performed the hematoxylin eosin (H&E) staining, and measured body weight, liver to body weight ratio and levels of ALT, AST, respectively. Histopathological analysis showed that extensive steatosis, liver cell cord derangement and intercellular spaces dilatation in EtOH-fed mice (**Figure 1A**). EtOH-fed mice and CD-fed mice reduced body weight in the early stages. Then EtOH-fed mice remained almost no change whereas the CD-fed mice slightly increased after the adaptive phase (**Figure 1B**). But the liver to body weight ratio in the EtOH-fed mice was higher than CD-fed mice at the end of model building (**Figure 1C**). Furthermore, ALT and AST enzymatic assay revealed significantly higher ALT and AST activity in serum compared to CD-fed mice (**Figure 1D**). Thus, alcohol feeding caused liver injury.

**FIGURE 2:**
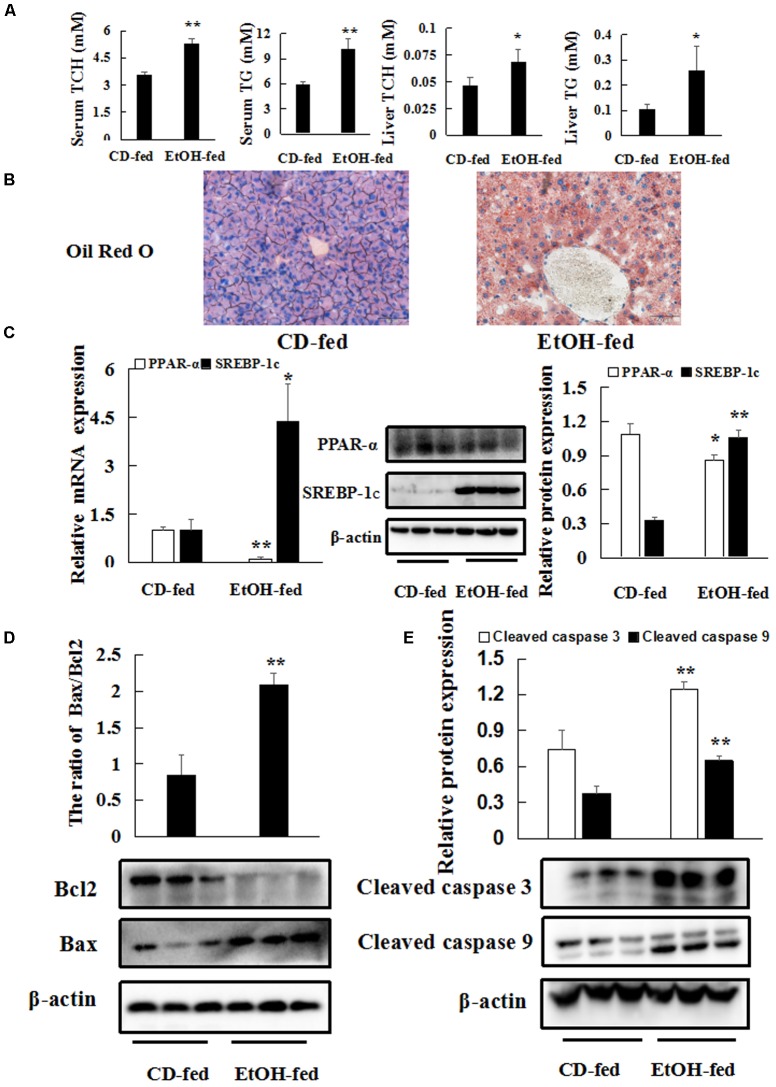
Chronic ethanol consumption produces hepatic steatosis and apoptosis. **(A)** Triglyceride (TG) and total cholesterol (TCH) levels in serum and liver tissues. **(B)** Representative Oil Red O staining of liver tissues 400×. **(C)** The protein and mRNA levels of PPAR-α and SREBP-1c in liver tissues. **(D,E)** The protein levels of Bax/Bcl2, cleaved caspase 3 and cleaved caspase9 in liver tissues. β-actin served as a loading control. The values represent means ± SD (*n* = 6 in CD-fed group, *n* = 6 in EtOH-fed group). ^∗^*P* < 0.05, ^∗∗^*P* < 0.01 vs. CD-fed group.

Next, we investigated the characteristic of hepatic steatosis and apoptosis in the progression of EtOH-induced liver injury. Hepatic steatosis is the most representative and prominent pathological feature of EtOH-induced liver injury. Herein, hepatic lipid contents were evaluated. As shown in **Figure 2A**, there was a dramatic increase in serum TG and TCH levels in EtOH-fed mice. Similarly, liver TCH and TG levels were also markedly increased in EtOH-fed mice, respectively, compared to CD-fed mice. Furthermore, Oil Red O staining of liver tissues indicated that ethanol consumption augmented the number of lipid droplets (**Figure 2B**). In addition, western blot and real-time PCR analyses showed that alcohol administration impressively enhanced SREBP-1c and Fasn expression but weakened PPAR-α expression at both mRNA and protein levels (**Figure 2C** and **Supplementary Figures S1A,B**). Furthermore, liver tissues in EtOH-fed mice showed a significant increase in the mRNA levels of Acaca compared with CD-fed mice (**Supplementary Figure S1B**). Then, the expression of protein Bax/Bcl2, cleaved caspase 3 and cleaved caspase 9 were up elevated in EtOH-fed mice compared to CD-fed mice (**Figures 2D,E**). All above data suggests that chronic ethanol feeding induce hepatic steatosis and apoptosis.

### Chronic Ethanol Consumption Induces MEG3 Expression in Hepatocytes *in Vivo* and *in Vitro*

**FIGURE 3:**
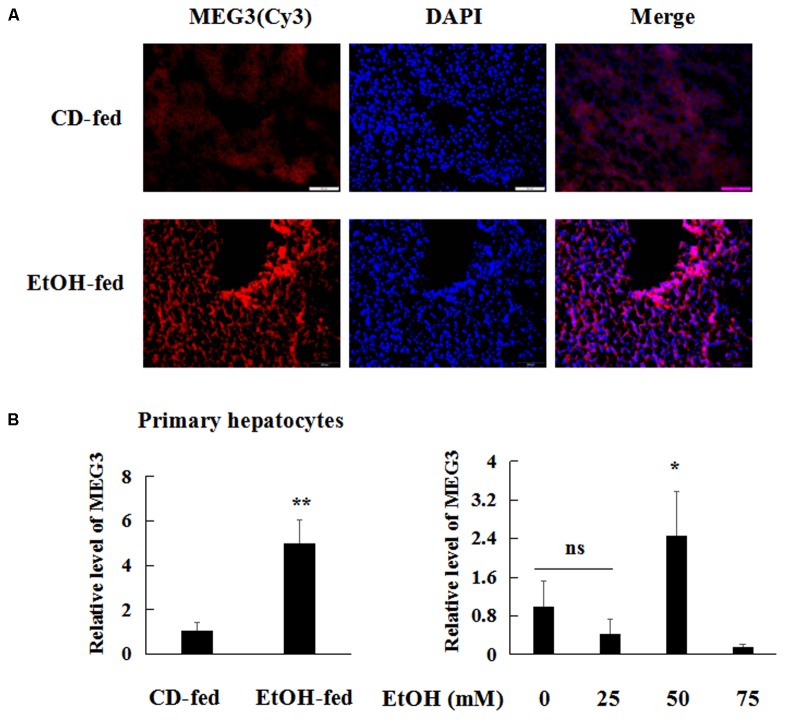
MEG3 was up-regulated in EtOH-fed mice and AML-12 cells. **(A)** ISH with anti-MEG3 probe in liver tissues 200×. **(B)** Real-time PCR analyses expression of MEG3 in primary hepatocytes and AML-12 cells. The values represent means ± SD. The data are representative of at least three independent experiments. ^∗^*P* < 0.05, ^∗∗^*P* < 0.01 vs. CD-fed group or control.

To identify the changes of MEG3 expression profile between EtOH-fed mice and CD-fed mice, FISH (ISH with anti-MEG3 probe) on liver tissues was performed. FISH analysis revealed MEG3 induction in EtOH-fed mice (**Figure 3A**). Moreover, real-time PCR showed that the level of MEG3 was elevated in the mouse primary hepatocytes (**Figure 3B**). Similar results were obtained in AML-12 cells (**Figure 3B**).

### Reducing MEG3 Expression Inhibits EtOH-Induced Hepatic Steatosis and Apoptosis

**FIGURE 4:**
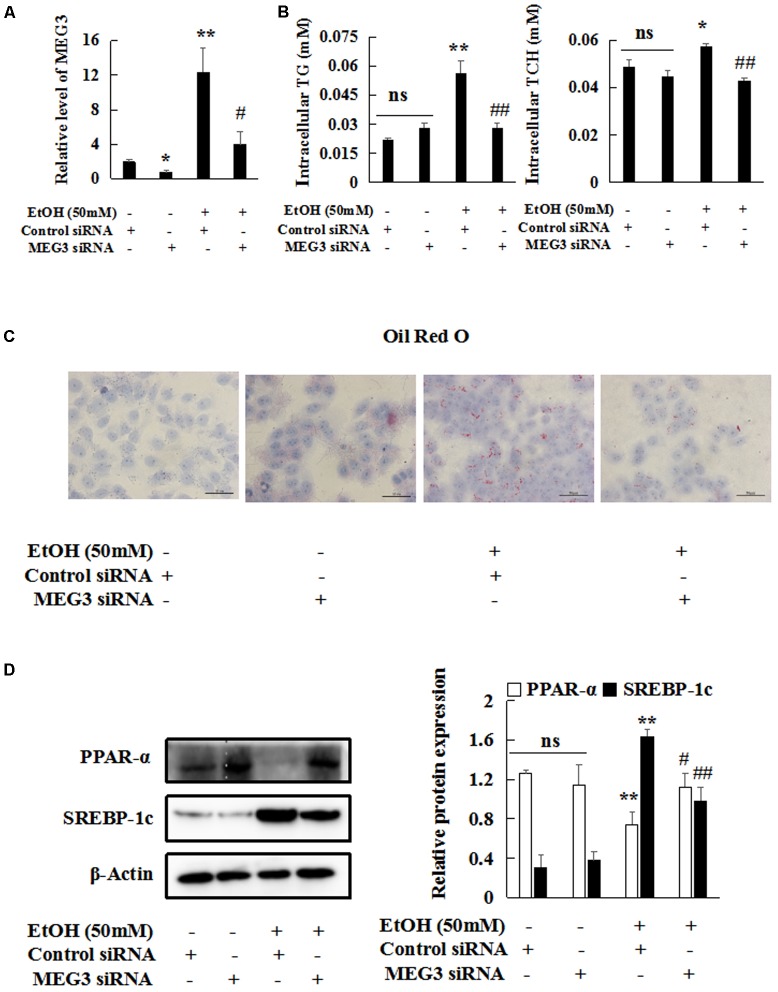
Reducing MEG3 expression inhibits EtOH-induced hepatic steatosis. **(A)** MEG3 successful knock down was verified by real-time PCR. **(B)** The levels of TG and TCH in AML-12 cells. **(C)** Oil Red O staining of AML-12 cells 400×. **(D)** Western blot analyses of PPAR-α and SREBP-1c in AML-12 cells. β-actin served as a loading control. Data shown are the mean ± SD from three independent experiments. ^∗^*P* < 0.05, ^∗∗^*P* < 0.01 vs. control. ^#^*P* < 0.05, ^##^P < 0.01 vs. EtOH-induced group.

To investigate the possible functional role of MEG3 in hepatocytes, MEG3 siRNA was transiently transfected into AML-12 cells without or with the treatment of 50 mM EtOH *in vitro*. The result of real-time PCR showed that the expression of MEG3 was decreased in MEG3 siRNA group compared with control siRNA (**Figure 4A**). Firstly, we investigated the effects of MEG3 on EtOH-induced hepatic steatosis. Intracellular TCH and TG levels were decreased in MEG3 siRNA compared to control siRNA (**Figure 4B**). The number of lipid droplet was significantly reduced in MEG3 siRNA group by Oil Red O staining in AML-12 cells (**Figure 4C**). Furthermore, the protein levels of SREBP-1c and Fasn were decreased in MEG3 siRNA but reduced the expression of PPAR-α protein compared with the control siRNA in EtOH-induced AML-12 cells (**Figure 4D** and **Supplementary Figure S1C**). Similarly, real-time PCR analysis showed that Fasn and Acaca were reduced in siMEG3 group (**Supplementary Figure S1D**). These observations suggest that MEG3 siRNA prevents EtOH-induced hepatic Steatosis.

**FIGURE 5:**
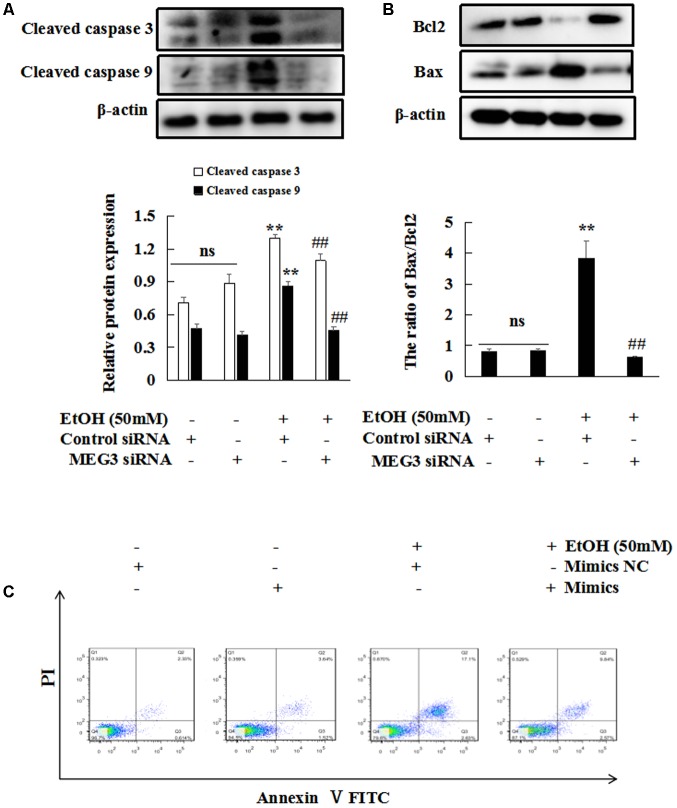
Reducing MEG3 expression inhibits EtOH-induced hepatic apoptosis. **(A,B)** Effect of MEG3 on expression of protein Bax/Bcl2, cleaved caspase 3 and cleaved caspase 9 in EtOH-induced AML-12 cells. The expression of proteins were determined by western blot. β-actin served as a loading control. **(C)** Flow cytometry analyses of apoptosis. The abscissa represents FITC-Annexin V staining and the ordinate represents PI staining. Data shown are the mean ± SD from three independent experiments. ^∗^*P* < 0.05, ^∗∗^*P* < 0.01 vs. control. ^#^*P* < 0.05, ^##^*P* < 0.01 vs. EtOH-induced group.

Next, we investigated the effects of MEG3 on EtOH-induced hepatic apoptosis. The protein expression of Bax/Bcl-2, cleaved caspase 3 and cleaved caspase 9 were decreased only in MEG3 siRNA group but not in control siRNA group in EtOH-induced AML-12 cells (**Figures 5A,B**). In addition, FITC-labeled Annexin V/PI staining showed an increase in the number of apoptotic hepatocytes in EtOH-treated group but a decrease in the number of apoptotic hepatocytes in MEG3 siRNA group (**Figure 5C**).

### MEG3 Is a Target of miR-let-7c-5p in EtOH-Induced Liver Injury

**FIGURE 6:**
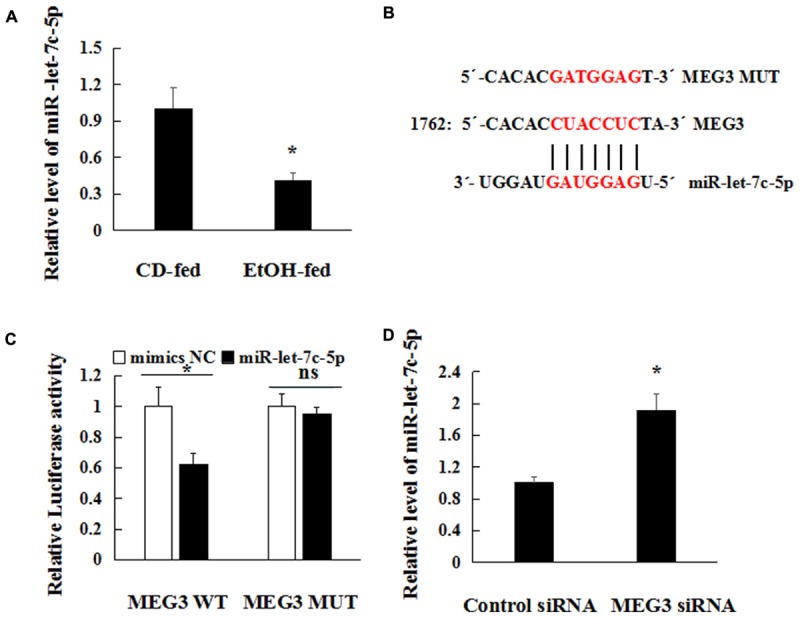
MEG3 is a target of miR-let-7c-5p in EtOH-induced Hepatic Injury. **(A)** miR-let-7c-5p relative level in primary hepatocytes isolated from the liver by real-time PCR. **(B)** The predicted positions of miR-let-7c-5p binding site on the MEG3. **(C)** Luciferase reporter assay in 293T cells were co-transfected with the MEG3-WT or mutant reporter vector in the miR-let-7c-5p binding sites and miR-let-7c-5p mimics. **(D)** Real-time PCR analysis of miR-let-7c-5p expression in AML-12 cells and transfected with MEG3 siRNA or control siRNA. Data shown are the mean ± SD from three independent experiments. ^∗^*P* < 0.05, ^∗∗^*P* < 0.01 vs. control or CD-fed group.

Recently, a range of evidence showed that lncRNAs have been reported to act as decoys to sequester miRNAs to prevent them from binding to targets (Quinn and Chang, 2016). As shown in **Figure 6A**, significant down-regulation of miR-let-7c-5p was observed in EtOH-fed mice by real-time PCR compared to CD-fed mice. To explore the underlying mechanisms of MEG3-mediated repression of miR-let-7c-5p, analysis with bioinformatic tools indicated that MEG3 and miR-let-7c-5p contain complementary base pairs (**Figure 6B**). To confirm this observation, wild-type (WT)-MEG3 luciferase reporter gene vector and mutant (Mut)-MEG3 vector were constructed. As illustrated in **Figure 6C**, the luciferase activity was significantly suppressed by the co-transfection of miR-let-7c-5p mimics and WT-MEG3 in 293T cells. However, co-transfection of miR-let-7c-5p mimics and MEG3-Mut did not change the luciferase activity. Furthermore, we transfected MEG3 siRNA into AML-12 cells to validate the regulation between MEG3 and miR-let-7c-5p. After MEG3 siRNA transfection, miR-let-7c-5p expression was significantly reduced, compared with control siRNA (**Figure 6D**). These data indicated the negative regulation between MEG3 and miR-let-7c-5p in EtOH-induced liver injury.

### Overexpression of miR-let-7c-5p Inhibits EtOH-Induced Hepatic Steatosis and Apoptosis

**FIGURE 7:**
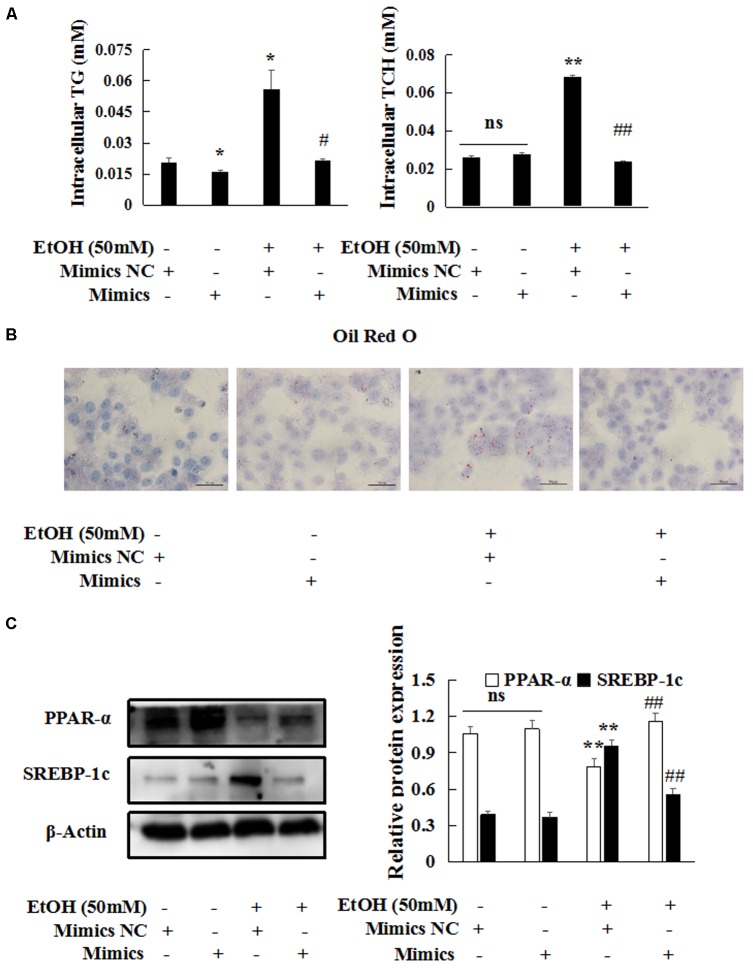
miR-let-7c-5p overexpression inhibits EtOH-induced hepatic steatosis. miR-let-7c-5p mimics or NC were transiently transfected into AML-12 cells, respectively. **(A–C)** Effects of miR-let-7c-5p on steatosis by oil red staining 400×, intracellular TCH and TG and western blot. β-actin served as a loading control. Data shown are the mean ± SD from three independent experiments. ^∗^*P* < 0.05, ^∗∗^*P* < 0.01 vs. control. ^#^*P* < 0.05, ^##^*P* < 0.01 vs. EtOH-treated group.

To clarify the function of miR-let-7c-5p in EtOH-induced hepatic steatosis and apoptosis, we transfected miR-let-7c-5p mimics into AML-12 cells. Overexpression of miR-let-7c-5p suppressed intracellular TG and TCH (**Figure 7A**) compared with mimics NC group. Oil Red O staining revealed that the number of lipid droplet was significantly decreased in miR-let-7c-5p mimics group (**Figure 7B**). Finally, the expression of protein PPAR-α was significantly induced, but expression of SREBP-1c and Fasn were reduced in miR-let-7c-5p mimics group compared with mimics NC group in EtOH-treated AML-12 cells (**Figure 7C** and **Supplementary Figure S1E**). Meanwhile, the mRNA levels of Fasn and Acaca were also decreased in miR-let-7c-5p mimics group compared with mimics NC group (**Supplementary Figure S1F**).

**FIGURE 8:**
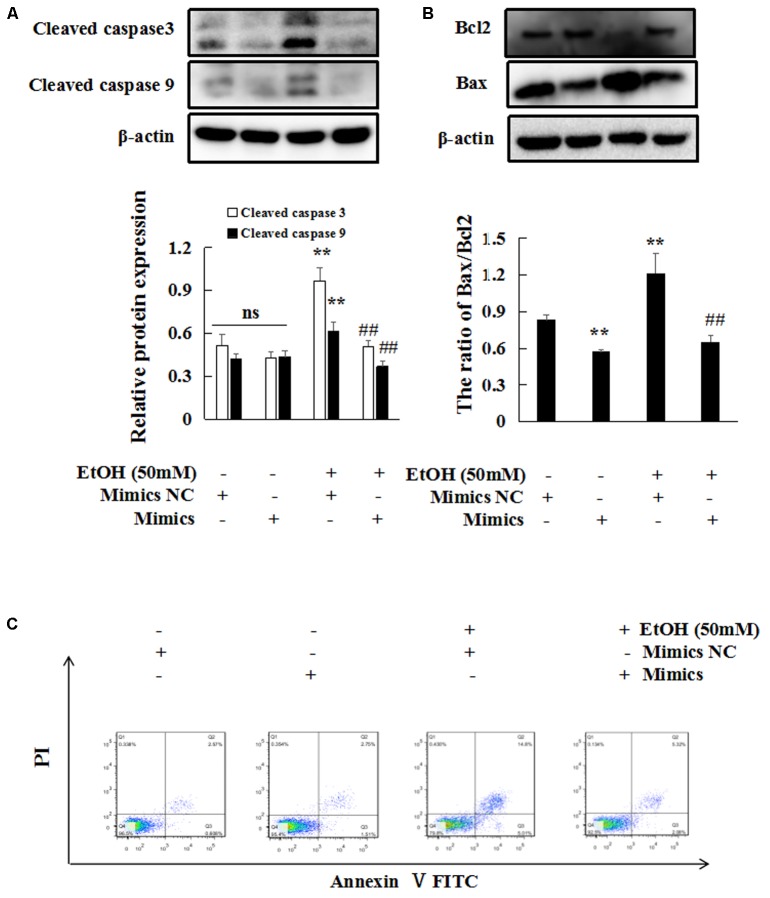
miR-let-7c-5p overexpression inhibits EtOH-induced hepatic apoptosis. **(A–C)** Effects of miR-let7c-5p on cell apoptosis by western blot and flow cytometry. β-actin served as a loading control. Data shown are the mean ± SD from three independent experiments. ^∗^*P* < 0.05, ^∗∗^*P* < 0.01 vs control. ^#^*P* < 0.05, ^##^*P* < 0.01 vs. EtOH-treated group.

Then, flow cytometry and western blot analyses were to explore the effects of miR-let-7c-5p on EtOH-induced hepatic apoptosis. EtOH-induced cleaved caspase 3, cleaved caspase 9 and Bax/Bcl2 protein expression were blocked by miR-let-7c-5p mimics (**Figures 8A,B**). Flow cytometric analysis showed that miR-let-7c-5p mimics decreased the percentage of apoptosis (**Figure 8C**).

Taken together, these results indicate that the overexpression of miR-let-7c-5p reduce EtOH-induced hepatic apoptosis and steatosis *in vitro.*

### miR-let-7c-5p Directly Targets the NLRC5 Gene in EtOH-Induced Liver Injury

**FIGURE 9:**
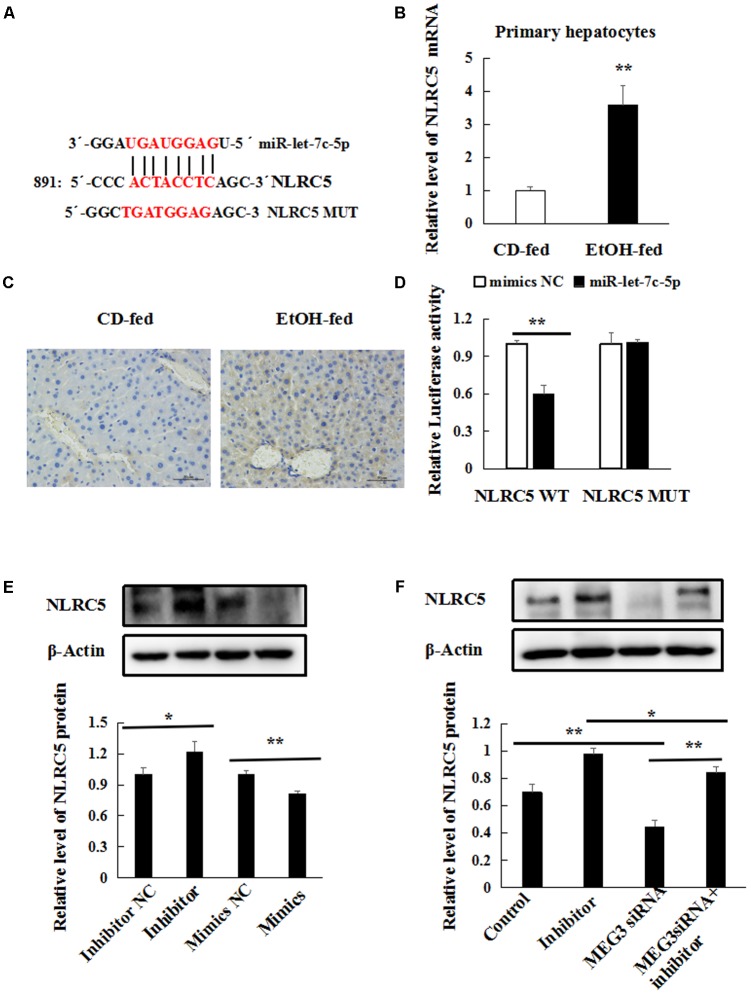
miR-let-7c-5p directly targets the NLRC5 gene in EtOH-induced liver injury. **(A)** Bioinformatics predicted miR-let-7c-5p binding sites in NLRC5 3′UTR. **(B)** Relative level of NLRC5 mRNA in primary hepatocytes by real-time PCR. **(C)** Immunohistochemical staining for NLRC5 in liver tissues 400×. **(D)** Luciferase reporter assay in 293T cells were co-transfected with the NLRC5-WT or mutant reporter vector in the miR-let-7c-5p binding sites and miR-let-7c-5p mimics. **(E)** Western blot analyses was performed to test NLRC5 expression after AML-12 cells were transfected with miR-let-7c-5p inhibitor NC, inhibitor, mimics NC or mimics. **(F)** Western blot analyses was performed to test NLRC5 expression. AML-12 cells were transfected with miR-let-7c-5p inhibitor or co-transfected with miR-let7c-5p inhibitor and the MEG3 siRNA. β-actin served as a loading control. Data shown are the mean ± SD from three independent experiments. ^∗^*P* < 0.05; ^∗∗^*P* < 0.01.

To identify the main target genes of miR-let-7c-5p, the Targetscan^1^ and miRanda^2^ algorithms were used to predicted target genes. NLRC5 was predicted to be a target of miR-let-7c-5p (**Figure 9A**). Then, immunohistochemistry (IHC) analysis and real-time PCR on liver tissues and mouse primary hepatocytes between the two groups were performed. NLRC5 highly expressed in EtOH-fed mice (**Figures 9B,C**). Furthermore, dual-luciferase reporter assay showed that the luciferase activity was significantly reduced by the co-transfection of miR-let7c mimics and NLRC5-Wt, meanwhile, co-transfection of miR-let-7c-5p mimics and NLRC5-Mut did not change the luciferase activity (**Figure 9D**). Consistent with the reporter assay, NLRC5 protein expression was decreased in miR-let-7c-5p mimics (**Figure 9E**). Conversely, NLRC5 protein increased after treatment with miR-let-7c-5p inhibitor (**Figure 9E**). Next we investigated the effect of MEG3 and miR-let-7c-5p co-processing on NLRC5, AML-12 cells was co-transfected with MEG3 siRNA and miR-let-7c-5p inhibitor, then the protein level of NLRC5 was determined by western blot. Results showed that miR-let-7c-5p inhibitor significantly increased the levels of NLRC5 protein, but MEG3 siRNA exerted an opposite function on NLRC5 expression. Moreover, the promotive effect of miR-let-7c-5p inhibitor on NLRC5 could be partially reversed by MEG3 siRNA (**Figure 9F**). These results indicate that NLRC5 is a direct target of miR-let-7c-5p and MEG3 regulates NLRC5 expression in miR-let-7c-5p-dependent manner.

## Discussion

Increasing studies have demonstrated that hepatic steatosis and apoptosis are key events during the progression of EtOH-induced liver injury. It is extremely crucial to discover the mechanism of hepatic steatosis and apoptosis. Recently, lncRNAs have been identified as novel regulators of the transcriptional and epigenetic networks. However, the role of lncRNA in EtOH-induced hepatic steatosis and apoptosis remains largely unknown. MEG3 has been reported to be involved in the epigenetic regulation of gene expression. Extensive functional studies have indicated that decreased of MEG3 occurs in many of human tumors and tumor derived cell lines, strongly suggesting that MEG3 inhibitors cancer progression (Greife et al., 2014; Li Z.H. et al., 2017; Terashima et al., 2017; Zhang J. et al., 2017). In addition, MEG3 as a novel regulator of BA homeostasis via PTBP1-mediated degradation of SHP mRNA (Zhang L. et al., 2017). Previous studies from our laboratory also have proved that MEG3 may play an important role in hepatic stellate cell activation and liver fibrosis progression (He et al., 2014). In the present study, MEG3 was up-regulated in EtOH-fed mice compared to CD-fed mice. More importantly, subcellular localization analysis of MEG3 by RNA fluorescence *in situ* hybridization assay confirmed cytosolic MEG3 induction in EtOH-fed mice. Recent studies proposed an viewpoint that lncRNA in cytoplasm may acts as a ceRNA to sequester miRNAs, as this lncRNA works like sponge in having miRNA exhausted, preventing mRNA from degradation (Tay et al., 2014; Chen, 2016). Therefore, these potential mechanisms of MEG3 may be concerned in alcoholic liver steatosis. In line with the up-regulation of MEG3 in EtOH-induced liver injury, treatment of AML-12 cells with EtOH resulted in a significant increase of MEG3 expression by real-time PCR. As results, knockdown of MEG3 inhibited hepatocyte steatosis and apoptosis in AML-12 cells, which is in agreement with previous reports that MEG3 promoted apoptosis (Liu et al., 2016a). It suggested that MEG3 may play a vital role in ethanol-induced hepatocyte steatosis and apoptosis. Therefore, more effects are needed to better elucidate molecular mechanisms of MEG3 in EtOH-induced liver Injury.

Recent studies suggest that lncRNAs may exert functions through targeting miRNAs (Quinn and Chang, 2016). It has been reported that miR-181b-12 could efficiently down-regulate MEG3 expression in brain nerve cells, then repressed its target mRNA (Liu et al., 2016a). A previous report showed that MEG3 acted as a ceRNA that protects PDCD4 mRNA from miR-21-mediated degradation (Chen et al., 2017). The present study revealed that miR-let-7c-5p was reduced in EtOH-fed mice, and it was negatively regulated by MEG3. The let-7 miRNA family was found to play an important role in human liver diseases such as biliary diseases (Glaser et al., 2014), alcoholic liver injury (McDaniel et al., 2017), hepatocellular carcinoma(Lan et al., 2011), hepatic fibrosis (Matsuura et al., 2016). What’s more, we found that miR-let-7c-5p mimics blocked the EtOH-induced hepatic steatosis and apoptosis in AML-12 cells.

Our results also indicated that NLRC5 was a direct target of miR-let-7c-5p and its expression was increased in EtOH-fed mice. Previous studies from our laboratory have proved that NLRC5 plays a negative role in ethanol-induced hepatic steatosis. We also demonstrated that NLRC5 promoted proliferation and activation of hepatic stellate cells during hepatic fibrosis (Xu et al., 2016). NLRC5 protein showed to be significantly reduced by miR-let-7c-5p mimics in AML-12 cells, whereas increased by miR-let-7c-5p inhibitor, suggesting that miR-let7c-5p could inversely regulate NLRC5 expression. We further confirmed that the existence of specific crosstalk between MEG3 and NLRC5 through competition for miR-let-7c-5p binding in AML-12 cells.

**FIGURE 10:**
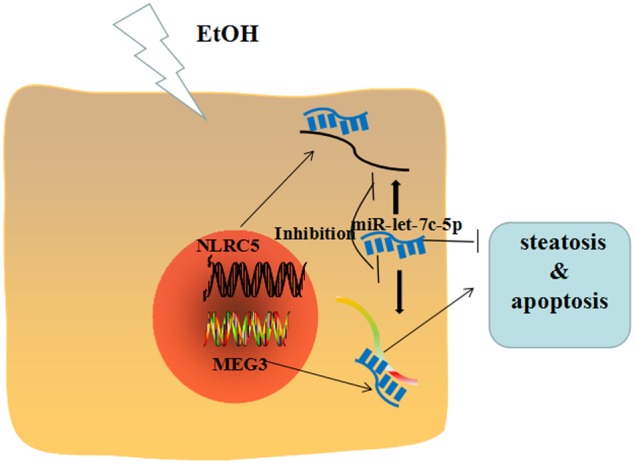
Overview of MEG3/miR-let-7c-5p/NLRC5 in EtOH-induced hepatic steatosis and apoptosis.

Taken together, our data indicate that the MEG3/miR-let-7c-5p/NLRC5 axis functions as an important player in EtOH-induced hepatic steatosis and apoptosis (**Figure 10**).

## Author Contributions

QW: performed all experiments, analyzed the data, and wrote the manuscript. ML, ZS, FB, HY, and XP: helped to perform animal experiments. YY, CH, and XM: participated in the design of the study. JL: conceived the study and revised the manuscript. All authors approved the final manuscript.

## Conflict of Interest Statement

The authors declare that the research was conducted in the absence of any commercial or financial relationships that could be construed as a potential conflict of interest. The reviewer XT and handling Editor declared their shared affiliation.

## Footnotes

1 http://www.targetscan.org

2 http://www.microrna.org

## Supplementary Material

FIGURE S1 **(A)** The protein levels of Fasn in liver tissues. **(B)** mRNA levels of Fasn and Acaca in liver tissues. Data shown are the mean ± SD, ^∗^*P* < 0.05; ^∗∗^*P* < 0.01. (*n* = 6 in CD-fed group, *n* = 6 in EtOH-fed group). **(C)** Western blot analyses of Fasn in AML-12 cells. **(D)** Real-time PCR analyses of Fasn and Acaca in AML-12 cells. **(E)** Western blot analyses of Fasn in AML-12 cells. **(F)** Real-time PCR analyses of Fasn and Acaca in AML-12 cells. β-actin served as a loading control. Data shown are the mean ± SD from three independent experiments. ^∗^*P* < 0.05, ^∗∗^*P* < 0.01 vs. control. ^#^*P* < 0.05, ^##^*P* < 0.01 vs. EtOH-treated group.
