# Supplementary figures and images for: The Long Non-coding RNA MEG3/miR-let-7c-5p Axis Regulates Ethanol-Induced Hepatic Steatosis and Apoptosis by Targeting NLRC5
Source: Front Pharmacol. 2018 Apr 10;9:302. doi: 10.3389/fphar.2018.00302 (PMC5902529; doi:10.3389/fphar.2018.00302)

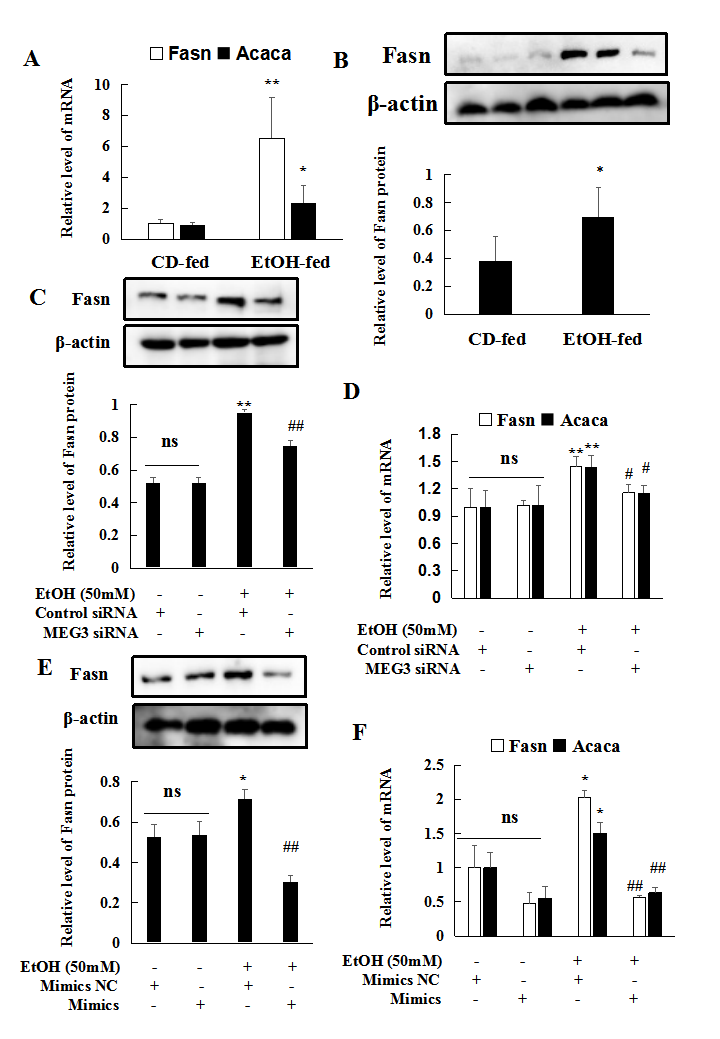

Supplement: FIGURE S1 — (A) The protein levels of Fasn in liver tissues. (B) mRNA levels of Fasn and Acaca in liver tissues. Data shown are the mean ± SD, ∗P < 0.05; ∗∗P < 0.01. (n = 6 in CD-fed group, n = 6 in EtOH-fed group). (C) Western blot analyses of Fasn in AML-12 cells. (D) Real-time PCR analyses of Fasn and Acaca in AML-12 cells. (E) Western blot analyses of Fasn in AML-12 cells. (F) Real-time PCR analyses of Fasn and Acaca in AML-12 cells. β-actin served as a loading control. Data shown are the mean ± SD from three independent experiments. ∗P < 0.05, ∗∗P < 0.01 vs. control. #P < 0.05, ##P < 0.01 vs. EtOH-treated group. [file Image_1.tif]
